# Supplementary material for: Splice-Junction-Based Mapping of Alternative Isoforms in the Human Proteome
Source: Cell Rep. Author manuscript; Available in PMC 2020 Jan 15. (PMC6961840; doi:10.1016/j.celrep.2019.11.026)
Supplement: 2 [file NIHMS1546469-supplement-2.zip › DF1/PXD006675/PulmonaryVein/PulmonaryVein_7_TTN_SNCTVSVHVSEPPR.pdf]

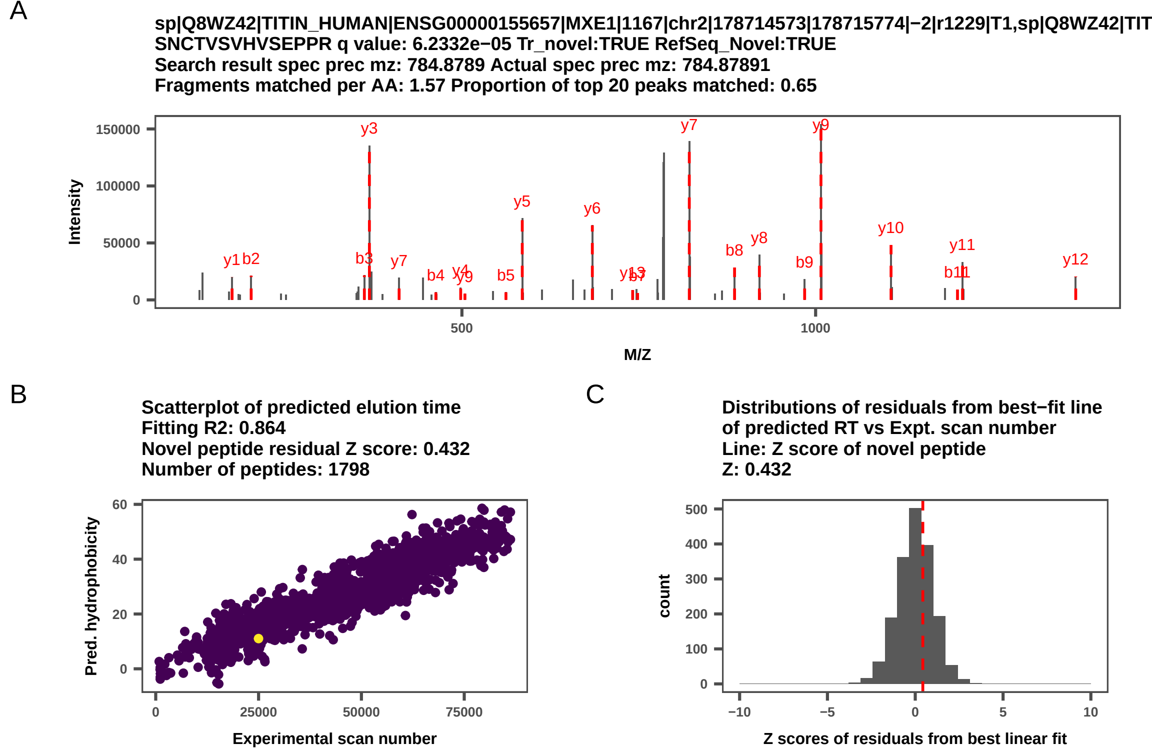
A
sp|Q8WZ42|TITIN_HUMAN|ENSG00000155657|MXE1|1167|chr2|178714573|178715774|−2|r1229|T1,sp|Q8WZ42|TIT
SNCTVSVHVSEPPR q value: 6.2332e−05 Tr_novel:TRUE RefSeq_Novel:TRUE
Search result spec prec mz: 784.8789 Actual spec prec mz: 784.87891
Fragments matched per AA: 1.57 Proportion of top 20 peaks matched: 0.65
Intensity
150000
100000
50000
0
y3
y7
y9
y5
y6
y10
y8
b8
b9
y11
y12
b11
y1
b2
b3
y7
b4
y4
y9
b5
y13
b7
500
1000
M/Z
B
Scatterplot of predicted elution time
Fitting R2: 0.864
Novel peptide residual Z score: 0.432
Number of peptides: 1798
Pred. hydrophobicity
60
40
20
0
0
25000
50000
75000
Experimental scan number
C
Distributions of residuals from best−fit line
of predicted RT vs Expt. scan number
Line: Z score of novel peptide
Z: 0.432
count
500
400
300
200
100
0
−10
−5
0
5
10
Z scores of residuals from best linear fit
